# Case Report: Diagnosis of Primary Cutaneous Amyloidosis Using Dermoscopy and Reflectance Confocal Microscopy

**DOI:** 10.3389/fmed.2020.619907

**Published:** 2021-01-21

**Authors:** Xiuli Wang, Hui Wang, Zhenyu Zhong, Liyun Zheng, Yifan Wang, Ze Guo, Hui Li, Min Gao

**Affiliations:** ^1^Department of Dermatology, The First Affiliated Hospital of Anhui Medical University, Hefei, China; ^2^Key Laboratory of Dermatology (Anhui Medical University), Ministry of Education, Hefei, China; ^3^Inflammation and Immune Mediated Diseases Laboratory of Anhui Province, Hefei, China

**Keywords:** primary cutaneous amyloidosis, dermoscopy, reflectance confocal microscopy, non-invasive technique, diagnosis

## Abstract

The dermoscopy and reflectance confocal microscopy (RCM) can provide new insights for diagnosis disease as non-invasive and easy-to-use tool. We described the dermoscopy and RCM characteristics of two patients with primary cutaneous amyloidosis (PCA) respectively. The dermoscopy characteristics were as follows: brownish macules with brown or white centers surrounded by hyperpigmented blotches, and a whitish scar-like center encircled by irregular brownish hyperpigmented spots or patches. The RCM features were increased melanin deposition in the basal layer, highly refractive structures with various shapes in the enlarged papillary dermis, and the increased pleomorphic structure of the dermal papillary ring. This is the first report the dermoscopy and RCM characteristics of PCA. We hope the characteristic dermoscopy and RCM appearances would provide a basis for doctors to diagnose and intervene earlier.

## Introduction

Primary cutaneous amyloidosis (PCA) is a relatively common disease, but it can cause physical and psychological distress due to itch and potentially disfiguring disorder. There are different subtypes of PCA, including macular amyloidosis, lichen amyloidosis, nodular amyloidosis, or a mixed appearance of macular amyloidosis and lichen amyloidosis, which is described as biphasic amyloidosis (1, 2). We observed two patients with PCA through dermoscopy and reflectance confocal microscopy (RCM). One of them was confirmed by histopathological examination, and the lesions presented as biphasic amyloidosis. Another patient was clinically diagnosed as macular amyloidosis.

Dermoscopy has been proved to be an important noninvasive technique to diagnose cutaneous pigmented diseases, which can magnify the pigmented structures of the epidermis, the dermo-epidermal junction, and the dermis (3, 4). RCM is a valuable, widely used tool for its noninvasive, dynamic real-time and high cellular resolution characteristic (5). These two tools are particularly suitable for disease screening before the histopathological examination, which may avoid some surgical operation.

## Case Report

**Figure 1:**
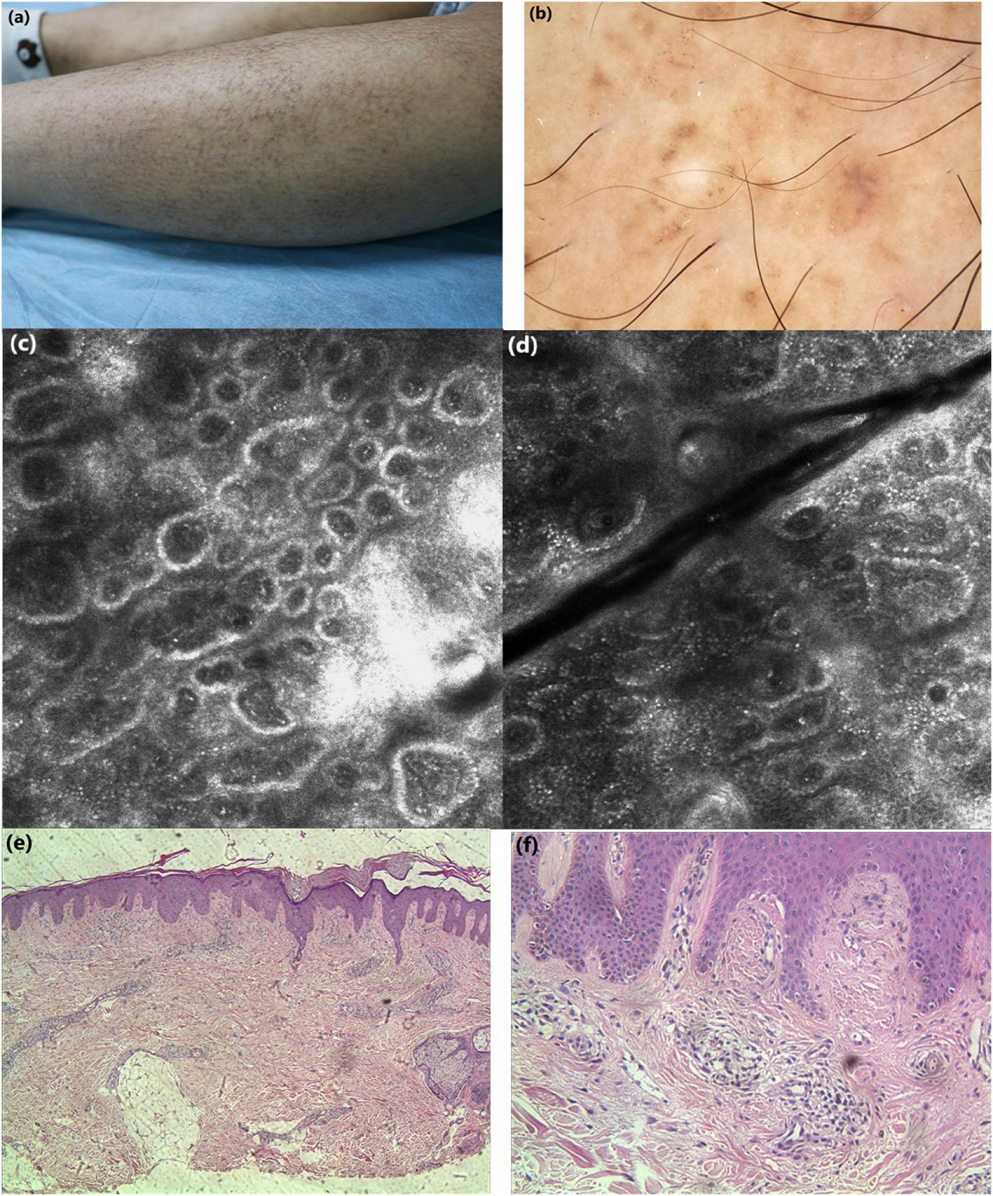
**(a)** Clinical features: macular hyperpigmentation in a rippling pattern and hyperkeratotic brownish papules. **(b)** Under dermoscopic: irregular brownish macules with brown centers surrounded by hyperpigmented blotches, whitish scar-like center encircled by brownish hyperpigmented patches. **(c,d)** Under RCM: hyperkeratosis, melanin deposition in the basal layer, highly refractive structures inside the enlarged papillary dermis in cloud-like agglomerate shape, some round moderately-highly bright round cells in dermis. **(e, f)** Histopathology: hyperkeratosis in the epidermis, increased melanin deposition in the basal layer, focal deposition of homogeneous acidophilic substance in dermal papilla (H&E stain).

**Figure 2:**
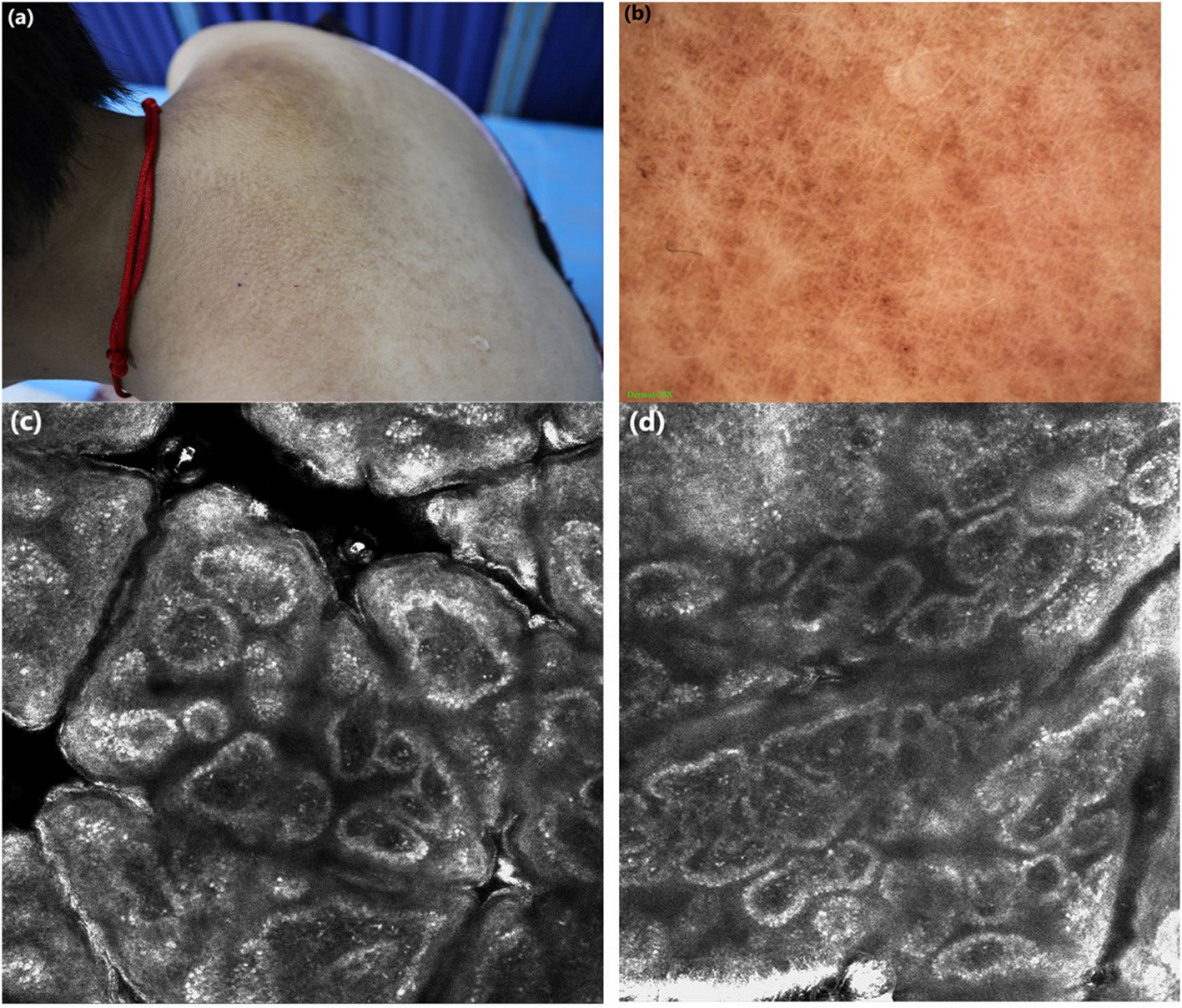
**(a)** Clinical features: brownish macules fused in a grid pattern. **(b)** Under dermoscopic: white center encircled by hyperpigmented blotches, other pigmented patches were partially fused. **(c,d)** Under RCM: highly refractive structures with various shapes (cloud-like agglomerate, dotted substance, coliform substance) gathered in the enlarged papillary dermis, the increased pleomorphic structure of the dermal papillary ring.

Case 1: A 25-years-old woman presented with hyperpigmented macules and maculopapular rash on her lower limbs for 4 years. The lesions were located mainly in the lower legs, which progressed slowly to the thighs and arms, with moderate pruritus. On physical examination, macular hyperpigmentation in a rippling pattern were observed over her limbs, and hyperkeratotic brownish papules in some areas. The patient was otherwise well, with no other skin symptoms or extracutaneous discomfort. The initial clinical diagnosis was consistent with PCA changes, which was more in line with biphasic amyloidosis (because the lesions had both the characteristics of macular amyloidosis and lichen amyloidosus) (Figure 1a). Case 2: A 65-years-old woman presented with a 10-year history of tan macules and papules on her back, with a medical history of cardiac disease. The lesions were persistent itching, without other skin discomfort. Physical examination revealed brownish macules fused in a grid pattern. We diagnosed it as macular amyloidosis (Figure 2a).

Case 1: Under dermoscopy, the appearance of some smaller brownish macules presented as irregular brown centers with small brown stripes, surrounded by hyperpigmented blotches, while other macules showed irregular inhomogeneous hyperpigmented blotches. The dermoscopic features of hyperkeratotic brownish papules displayed a whitish scar-like center encircled by irregular brownish hyperpigmented spots or patches (Figure 1b). Case 2: On dermoscopic examination, the macula lesions showed white center encircled by hyperpigmented blotches, while other pigmented patches were partially fused (Figure 2b).

Case 1: Upon RCM, the examination displayed the hyperkeratosis in the epidermis, the increased melanin deposition in the basal layer, the highly refractive structures with cloud-like agglomerate, and dotted substance inside papillary dermis. Part of the dermal papillary rims were obscured, surrounded by some round, moderately-highly bright round cells (Figures 1c,d). Case 2: Under RCM, the appearance of PCA were the highly refractive structures with various shapes (cloud-like agglomerate, dotted substance, coliform substance, and so on) gathered in the enlarged papillary dermis, and the increased pleomorphic structure of the dermal papilla ring (Figures 2c,d).

Histopathological examination of case 1 showed hyperkeratosis in the epidermis, epidermal hyperplasia, downward extended of part of the epidermis, increased melanin deposition in the basal layer, and focal deposition of homogeneous acidophilic substance in the enlarged dermal papilla (Figures 1e,f).

## Conclusion

We presented two patient with PCA, and to our knowledge, this is the first report the dermoscopy and RCM characteristics of PCA. The dermoscopic findings of macular amyloidosis often showed a common feature, that is a white or brown central hub surrounded by brownish pigmentation (6). Besides, a white central hub or whitish scar-like center surrounded by pigmentation were often presented in the lichen amyloidosis (6). The RCM features of PCA have not been adequately described in the literature. Only two articles have been published to report the RCM features of PCA (7, 8). Upon RCM, the characteristics of increased melanin in the basal cell layer, cloud-like agglomerate in the dermal papilla, and melanophage infiltrating in the layer of superficial dermis, which were corresponding to histopathological findings: basal hyper pigmentation, amyloid deposition, pigment incontinence (8).

PCA is usually diagnosed clinically. But for cases with atypical presentation, the noninvasive technique, dermoscopy combined with RCM, can be used as a reference for clinicians to diagnose PCA. On our observation, the dermoscopy features were brownish macules with brown or white centers surrounded by hyperpigmented blotches, and a whitish scar-like center encircled by irregular brownish hyperpigmented spots or patches. The RCM features were increased melanin deposition in the basal layer, highly refractive structures with various shapes (cloud-like agglomerate, dotted substance, coliform substance and so on) in the enlarged papillary dermis, and the increased pleomorphic structure of the dermal papilla. We hope the characteristic dermoscopy and RCM appearances would provide a basis for doctors to diagnose and intervene earlier.

## Data Availability Statement

The original contributions presented in the study are included in the article/supplementary material, further inquiries can be directed to the corresponding author/s.

## Ethics Statement

The studies involving human participants were reviewed and approved by Ethical Review Committee of Anhui Medical University. The patients/participants provided their written informed consent to participate in this study. Written informed consent was obtained from the individual(s) for the publication of any potentially identifiable images or data included in this article.

## Author Contributions

All authors listed have made a substantial, direct and intellectual contribution to the work, and approved it for publication.

## Conflict of Interest

The authors declare that the research was conducted in the absence of any commercial or financial relationships that could be construed as a potential conflict of interest.
